# Hydrogen: Potential Applications in Solid Organ Transplantation

**DOI:** 10.1155/2021/6659310

**Published:** 2021-11-24

**Authors:** Fuxun Yang, Yu Lei, Rongan Liu, Xiaoxiu Luo, Jiajia Li, Fan Zeng, Sen Lu, Xiaobo Huang, Yunping Lan

**Affiliations:** Department of ICU, Sichuan Provincial People's Hospital, University of Electronic Science and Technology of China, Chengdu, China

## Abstract

Ischemia reperfusion injury (IRI) in organ transplantation has always been an important hotspot in organ protection. Hydrogen, as an antioxidant, has been shown to have anti-inflammatory, antioxidant, and antiapoptotic effects. In this paper, the protective effect of hydrogen against IRI in organ transplantation has been reviewed to provide clues for future clinical studies.

## 1. Introduction

Ischemia reperfusion injury (IRI) is one of the most common clinical complications of organ transplantation [1]. The damage mechanism involves cell ion changes [2], mitochondrial metabolism [3], reactive oxygen species (ROS) system activation [4, 5], various inflammatory reactions [6, 7], and other pathophysiological changes. In severe cases, it may even cause primary graft dysfunction, prolong total hospital stay, and greatly increase mortality risk in solid organ transplant recipients [8–13]. Although various isolated organ protection platforms, such as ex vivo lung perfusion (EVLP) and LifePort Liver Transporter (LIFESPORT), have been developed clinically and extensive research and improvement have been achieved for organ preservation fluid [14–20], IRI cannot be completely prevented.

Graft ischemia leads to the harmful production of ROS; however, the reoxygenation process during reperfusion is the reason for the production of most ROS, activation of the complement system, and initiation of inflammatory responses [21]. Occlusion of vascular supply during transplantation leads to severe hypoxia of endothelial cells, which become an important source and target of ROS. Mitochondrial dysfunction, neutrophil initiation, xanthine oxidase, and NADPH oxidase play key roles in this process [22] In turn, excessive oxidizing agents lead to tissue damage and cell death by inducing the peroxidation of DNA, proteins, and lipids. Therefore, use of anti-ROS agents has been an important strategy for reducing IRI during organ transplantation.

Hydrogen is widely distributed in nature, with a concentration of 0.00006% in the air [23]. Under physiological states, human intestinal flora can produce a large amount of hydrogen, which participates in human physiological processes and is eventually discharged or metabolized from the lungs to produce nontoxic water [24]. Selective antioxidant function of hydrogen has been demonstrated in previous studies [25]; with the intensification of studies, hydrogen has been proved to exert several effects, such as anti-inflammatory [26–28], antioxidant [29, 30], and antiapoptosis effects [31, 32]. In recent years, the use of hydrogen has become an important part of the use of gases in medical treatments. Hydrogen has been used in various disease models and treatment studies, including IRI in solid organ transplantation. However, the specific mechanism of hydrogen in treating IRI in solid organ transplantation is not completely clear at present. Currently available experiments and studies have found that the mechanism may be related to its selective antioxidant effect and its ability to reduce inflammatory responses and inhibit cell apoptosis. The research progress of its application in solid organ transplantation is summarized below (Table 1).

### 1.1. Application of Hydrogen in Lung Transplantation

Primary reflective graft dysfunction caused by IRI is one of the common clinical complications of lung transplant recipients [33], with an incidence of up to 30%, which greatly increases the risk of death of lung transplant recipients [8]. Therefore, maintenance of lung function is crucial for these recipients [34]. Several studies have been conducted to determine the protective effect of hydrogen on lung transplantation, which is mainly reflected during the preexportation process of the donor lung, the cold ischemia period, and the transplantation process.

#### 1.1.1. Application of Hydrogen in the Donor Lung before Separation

Protection of the donor lung has always been imperative, and effective measures can expand the source of donors. Previous studies have found that inhalation of hydrogen by the donor can improve the compliance and oxygen index of the transplanted lung [35–37]. The possible mechanism occurs mainly through the following ways:

1. *Antioxidant Effect*. In a lung transplantation brain death rat model, 2% hydrogen for donor and recipient ventilation restrained malondialdehyde- and myeloperoxidase-mediated inhibition of heme oxygenase-1 and increased the activity of superoxide dismutase (sod) and other antioxidants [36, 37], to protect lung function.
2. *Antiapoptotic Effect*. In a cardiac and brain death animal model, 2–3% hydrogen inhalation could effectively regulate the expression of the antiapoptotic protein Bcl-2 and the proapoptotic protein Bax, thereby inhibiting the apoptotic process caused by Caspase-3 positive cells [35–37].
3. *Anti-inflammatory Effect*. In both the cardiac death model and brain death model, hydrogen inhalation could effectively reduce the expression level of proinflammatory factors such as IL-8, IL-6, and TNF-*α*, thus alleviating the lung injury of the donor before extracting the lung [35–37]. In addition, Tanaka et al. [38] sequenced hydrogen-pretreated transplant donors and found that hydrogen treatment induced the expression of proteins (including Clara cells) with anti-inflammatory and antioxidant effects and increased intracellular tissue adenosine triphosphate (ATP) and heat shock protein 70 (HSP70) expression levels. Hydrogen treatment also induced surfactants to regulate the expression of C/EBPA and C/EBPB transcription factors. The above gene changes provided effective clues for our later exploration of energy metabolism and surfactant-related pathways.

#### 1.1.2. Application of Hydrogen in the Cold Ischemia Period

The treatment of the lung in the cold ischemia period includes storage of organ protective fluid and the repair of the EVLP platform.

*(1) Application of Hydrogen in In Vitro Lung Organ Preservation Solution*. As an important part of donor protection for lung transplantation, the continuous optimization of organ preservation solution has always been a clinical hotspot. Hydrogen-rich organs preserved in liquid can also protect lungs in isolation. Masao et al. preserved donor lungs provided by canine or rat lung transplantation models in hydrogen-rich perfusion fluid. Compared with the control group, donor lungs maintained a higher oxygen partial pressure and had less perivascular edema in the transplanted lung [38]. Hydrogen-rich preservation solutions on the one hand can reduce the expression of proinflammatory cytokine (TNF-*α* and IL-1*β*) mRNA and on the other hand can inhibit the expression of 8-OHdG, which is an indicator of oxidative stress [39, 40]. This effect was demonstrated to be achieved through the Nrf2-HO-1 pathway. In addition, hydrogen can also improve the static P-V curve and histological score of the transplanted lung by expanding the donor lung infiltrated in the organ preservation solution [41]. Zhang et al. used pulmonary microvascular endothelial cells to simulate IRI in a lung transplantation model. This effect may be achieved by inhibition of the p38 mitogen-activated protein kinase (MAPK) and nuclear factor-kappaB (NF-*κ*B) pathways to achieve the objective of inflammatory injury of pulmonary microvascular endothelial cells (PMVECs) [42].

*(2) Application of Hydrogen in EVLP Platform*. In isolated lungs, currently, EVLP, as an important weapon for lung repair, has been applied more and more widely in clinical practice. As early as 2014, Entaro et al. found that a 2% hydrogen group significantly upregulated mitochondrial-related genes contributing to the lung during EVLP repair, such as heme oxygenase-1 (*HO-1*), peroxisome proliferator-activated receptor-gamma coactivator (*pgC-1α*), and nuclear respiratory factor-1 (*Nrf-1*). At the same time, the enzyme activities of mitochondrial complexes I and II and the activity of mitochondrial complex IV were significantly increased, suggesting that the protective effect of hydrogen on donor lung function may be due to the intervention of mitochondrial oxidative stress to achieve lung protection [43]. Subsequently, Haam's team demonstrated that the use of hydrogen as an intervention during EVLP resulted in a decreased pulmonary vascular resistance index, decreased expression of inflammatory factors such as IL-1*β*, IL-6, IL-8, and TNF-*α*, and significantly reduced apoptosis. At the same time, the phosphorylation of all MAPK-related enzymes in the hydrogen intervention group was low, suggesting that the changes of hydrogen during EVLP may be realized through the MAPK pathway [44]. Subsequent studies confirmed that the protective effect of hydrogen on the transplanted lung not only exists during EVLP, but also after donor transplantation [45].

*(3) Application of Hydrogen in Lung Transplantation Surgery*. Meng et al. [46] and Kawamura et al. [47] also applied hydrogen to lung transplantation in animal models and found that hydrogen could reduce lipid peroxidation of the graft, reduce the production of inflammatory factors such as IL-6, and significantly reduce the infiltration of macrophages in lung tissue. In the study of Kawamura et al., the mRNA of Bcl-2 and Bcl-XL related to apoptosis was significantly upregulated in the hydrogen group 2 h after ischemic reperfusion, and the proteins of Bcl-2 and Bcl-XL were increased 6 h after reperfusion, suggesting that the antiapoptotic effect of hydrogen plays an important protective role in the process of lung transplantation.

#### 1.1.3. Application of Hydrogen in Liver Transplantation

At present, the studies on the effect of hydrogen on ischemia/reperfusion injury in liver transplantation mainly focus on the organ preservation solution of the donor liver and during liver transplantation. In the animal model of IRI after liver transplantation, hydrogen-rich preservation solution on the one hand can improve the redox state of the donor liver [48, 49] and upregulate HO-1 expression by inhibiting the cytoplasmic MKK4-JNK-mediated cell death pathway [48], thus providing better function and morphological protection for the donor liver [50]. On the other hand, Shimada et al. found that reperfusion of the donor liver with hydrogen-rich preservation solution can protect mitochondrial function in the early stages and inhibit subsequent oxidative stress and the inflammatory cascade, thereby reducing liver reperfusion injury [51]. Continuous inhalation of 2% hydrogen for 1 h at the beginning of liver transplantation in animal models can regulate the protection of rat liver from ischemia/reperfusion injury by activating the NF-*κ*B signaling pathway [28].

#### 1.1.4. Application of Hydrogen in Kidney Transplantation

There are relatively few studies on hydrogen in kidney transplantation, most of which focus on the improvement of organ preservation fluid. Abe et al. suggested that hydrogen-rich UW solution reduced oxidative stress in renal grafts at the early stage and reduced renal tubular apoptosis and mesenchymal macrophage infiltration. Histopathologically, the treatment with hydrogen-rich UW fluid reduced renal tubular damage and inhibited the progression of interstitial fibrosis [52]. Kobayashi and Sano [53] facilitated kidney preservation in a dissolved hydrogen fluid infusion after transplantation. Renal blood flow could be detected in the experimental group six days after transplantation, and urine was detected in the bladder. These studies suggest the potential of hydrogen in kidney IRI, but this remains to be confirmed by more research.

#### 1.1.5. Application of Hydrogen in Small Intestinal Transplantation

Hydrogen also has antioxidant and anti-inflammatory effects in studies of small intestinal transplants. Shigema et al. and Yamamoto et al. used a noctilucent, hydrogen-rich solution for enteric perfusion of the transplanted intestine, which significantly inhibited the levels of oxidative indices, malondialdehyde, and 8-hydroxydeoxyguanosine [54, 55]. The levels of mRNA and protein of proinflammatory cytokines, such as inducible nitric oxide synthase and interleukin-6, were significantly inhibited in the hydrogen-rich solution group (HRGS). In the HRGS group, crypt cell apoptosis was significantly inhibited, and the villi in the small intestine were more complete [54, 55]. Buchholz et al. also demonstrated that hydrogen treatment alleviated intestinal IRI and improved survival by regulating the increased antioxidant capacity and myoglobin oxygenase-1 [56]. Heme oxygenase-1 is largely regulated by the redox sensitive transcription factor, the nuclear factor RBC-2-related factor 2 (Nrf2). Therefore, OH-1 may be explored as a target for future hydrogen studies on intestinal transplantation. The study also found that the hydrogen pretreatment increased gastrointestinal activity, improved the contractability of jejunal smooth muscle of intestinal grafts, inhibited mucosal erosion and exfoliation of a large number of epithelial cells, and maintained basic permeability [57]. These findings further support the idea that hydrogen treatment during small intestinal transplantation can maintain the integrity of the intestinal mucosa while maintaining gastrointestinal activity and reducing postoperative complications.

#### 1.1.6. Application of Hydrogen in Heart Transplantation

The main applications of hydrogen in IRI in heart transplantations are hydrogen inhalation treatment and hydrogen-rich water treatment. Current studies suggest that hydrogen-rich organ protective fluid or hydrogen inhalation pretreatment can regulate oxidative stress markers of the ischemic myocardium on the one hand, such as Box1 protein and 8-hydroxy-2′-deoxyguanosine (8-OHDG) in the serum high mobility group, and enhance the antioxidant capacity of myocardium tissue. On the other hand, hydrogen treatment can regulate the mRNA and protein expression levels of apoptosis-related molecules, such as proapoptotic molecules, Bax and Bcl-2, which can reduce apoptosis [58–60]. In the process of ischemia reperfusion in myocardial tissue, a large amount of ROS production will affect mitochondrial homeostasis. Mitochondria are the main sites of intracellular ROS production and also the target of ROS but produce energy for normal heart function and ATP for most cells. The use of hydrogen-rich organ protectors in heart transplants protects mitochondria and stimulates mitochondrial proliferation in heart transplants. Hydrogen-rich organ protective fluid activates ATP synthase and mitochondrial biogenetic genes and maintains ATP levels in transplanted tissues [59]. At the same time, mitochondria-related genes such as *PGC-1α*, *NRF-1*, and *PPAR-G* were significantly upregulated in the hydrogen-rich protective solution intervention group, and even the gene and protein expressions of HO-1 were upregulated. *PPAR-G* regulates HO-1 expression, and HO-1 activates mitochondria by promoting the expression of the *NRF-1* gene through nuclear factor erythroid 2-associated factor (NRF), suggesting that the protective effect of hydrogen-rich protective fluid may be achieved through the PPAR-*α*/HO-1 signaling pathway to protect the donor heart.

## 2. Conclusions

The graft protection effect of hydrogen has been gradually confirmed in basic research; however, the exact mechanism leading to these effects is still not fully understood. Nevertheless, these basic findings may provide clues for the use of hydrogen in the treatment of graft IRI. In the past, hydrogen was only given in the gaseous state, which obviously limited its clinical application. Currently, hydrogen delivery has developed into various forms, including liquid, gas, and solid, and has entered other clinical fields, so it is a strong prospect for clinical application. There are currently few studies on hydrogen and nonsolid organ transplantation, which may present a research direction for the future. Further animal studies and preliminary human clinical trials are needed to lay the groundwork for the clinical use of hydrogen as a drug in the near future.

## Figures and Tables

**Table 1:** Application of hydrogen in different organs.

Organ	Use-pattern	Time	Reference
Lung	2–3% hydrogen	Donor	[35–38, 41]
Lung	Hydrogen-rich solution	Cold ischemia phase	[39, 40, 48]
Lung	2% hydrogen	EVLP	[43, 45, 46]
Lung	3% hydrogen	PMVECs	[42]
Lung	3% hydrogen & CO	Cold ischemia phase	[46]
Lung	2% hydrogen	During lung transplantation	[47]
Liver	Hydrogen-rich solution	Cold ischemia phase	[48, 50]
Liver	Hydrogen flush after cold storage	Cold ischemia phase	[49]
Liver	Hydrogen-rich perfusion fluid	Cold ischemia phase	[51]
Kidney	Hydrogen-rich solution	Cold ischemia phase	[52, 53]
Small intestine	Hydrogen-rich solution	Cold ischemia phase	[54, 55]
Small intestine	Hydrogen-bubbled preservation solution	Cold ischemia phase	[56]
Small intestine	2% hydrogen	Perioperative period	[57]
Heart	1–3% hydrogen	1 h before and after reperfusion	[58]
Heart	Hydrogen-rich water bath	Cold ischemia phase	[59]
Heart	Hydrogen-rich solution	Cold ischemia phase	[60]

## Data Availability

No data were used to support this study.

## References

[1] Christie J. D., Edwards L. B., Kucheryavaya A. Y. (2011). The Registry of the International Society for Heart and Lung Transplantation: Twenty-eighth Adult Lung and Heart-Lung Transplant Report--2011. *The Journal of Heart and Lung Transplantation*.

[2] Allen D. G., Xiao X.-H. (2003). Role of the cardiac Na^+^/H^+^ exchanger during ischemia and reperfusion. *Cardiovascular Research*.

[3] Halestrap A. P., Clarke S. J., Javadov S. A. (2004). Mitochondrial permeability transition pore opening during myocardial reperfusion—a target for cardioprotection. *Cardiovascular Research*.

[4] Guan L.-Y., Fu P.-Y., Li P.-D. (2014). Mechanisms of hepatic ischemia-reperfusion injury and protective effects of nitric oxide. *World Journal of Gastrointestinal Surgery*.

[5] Reiter R. J., Acuña-Castroviejo D., Tan D. X., Burkhardt S. (2001). Free radical-mediated molecular Damage. *Annals of the New York Academy of Sciences*.

[6] Jaeschke H. (2003). Molecular mechanisms of hepatic ischemia-reperfusion injury and preconditioning. *American Journal of Physiology-Gastrointestinal and Liver Physiology*.

[7] Turer A. T., Hill J. A. (2010). Pathogenesis of myocardial ischemia-reperfusion injury and rationale for therapy. *The American Journal of Cardiology*.

[8] Porteous M. K., Diamond J. M., Christie J. D. (2015). Primary graft dysfunction. *Current Opinion in Organ Transplantation*.

[9] Wertheim J. A., Petrowsky H., Saab S., Kupiec-Weglinski J. W., Busuttil R. W. (2011). Major challenges limiting liver transplantation in the United States. *American Journal of Transplantation*.

[10] Debout A., Foucher Y., Trébern-Launay K. (2015). Each additional hour of cold ischemia time significantly increases the risk of graft failure and mortality following renal transplantation. *Kidney International*.

[11] Chapman J. R., O’Connell P. J., Nankivell B. J. (2005). Chronic renal allograft dysfunction. *Journal of the American Society of Nephrology*.

[12] Bennett-Guerrero E., Feierman D. E., Barclay G. R. (2001). Preoperative and intraoperative predictors of postoperative morbidity, poor graft function, and early rejection in 190 patients undergoing liver transplantation. *Archives of Surgery*.

[13] Diamond J. M., Lee J. C., Kawut S. M. (2013). Clinical risk factors for primary graft dysfunction after lung transplantation. *American Journal of Respiratory and Critical Care Medicine*.

[14] Yeung J. C., Krueger T., Yasufuku K. (2017). Outcomes after transplantation of lungs preserved for more than 12 h: a retrospective study. *The Lancet Respiratory Medicine*.

[15] Cypel M., Yeung J. C., Liu M. (2011). Normothermic ex vivo lung perfusion in clinical lung transplantation. *New England Journal of Medicine*.

[16] Bellini M. I., Nozdrin M., Yiu J., Papalois V. (2019). Machine perfusion for abdominal organ preservation: a systematic review of kidney and liver human grafts. *Journal of Clinical Medicine*.

[17] Jing L., Yao L., Zhao M., Peng L.-P., Liu M. (2018). Organ preservation: from the past to the future. *Acta Pharmacologica Sinica*.

[18] Kaths J. M., Paul A., Robinson L. A., Selzner M. (2018). Ex vivo machine perfusion for renal graft preservation. *Transplantation Reviews*.

[19] Büyük B., Demirci T., Adalı Y., Eroğlu H. A. (2019). A new organ preservation solution for static cold storage of the liver. Amniotic fluid1. *Acta Cirurgica Brasileira*.

[20] Kolonko A., Król R., Chudek J., Skrzypek M., Cierpka L., Więcek A. (2020). Early graft function and intrarenal resistant index after kidney transplantation using Biolasol—a new solid organ preservation fluid. *Artificial Organs*.

[21] Akhtar M., Henderson T., Sutherland A., Vogel T., Friend P. (2013). Novel approaches to preventing ischemia-reperfusion injury during liver transplantation. *Transplantation Proceedings*.

[22] Panieri E., Santoro M. M. (2015). ROS signaling and redox biology in endothelial cells. *Cellular and Molecular Life Sciences*.

[23] Zheng X. F., Sun X. J., Xia Z. F. (2011). Hydrogen resuscitation, a new cytoprotective approach. *Clinical and Experimental Pharmacology and Physiology*.

[24] Sano M., Suzuki M., Homma K. (2018). Promising novel therapy with hydrogen gas for emergency and critical care medicine. *Acute Medicine & Surgery*.

[25] Ohsawa I., Ishikawa M., Takahashi K. (2007). Hydrogen acts as a therapeutic antioxidant by selectively reducing cytotoxic oxygen radicals. *Nature Medicine*.

[26] Sun Q., Cai J., Liu S. (2011). Hydrogen-rich saline provides protection against hyperoxic lung injury. *Journal of Surgical Research*.

[27] Xie K., Liu L., Yu Y., Wang G. (2014). Hydrogen gas presents a promising therapeutic strategy for sepsis. *BioMed Research International*.

[28] Zhang C.-B., Tang Y.-C., Xu X.-J., Guo S.-X., Wang H.-Z. (2015). Hydrogen gas inhalation protects against liver ischemia/reperfusion injury by activating the NF-*κ*B signaling pathway. *Experimental and Therapeutic Medicine*.

[29] Huang C.-S., Kawamura T., Peng X. (2011). Hydrogen inhalation reduced epithelial apoptosis in ventilator-induced lung injury via a mechanism involving nuclear factor-kappa B activation. *Biochemical and Biophysical Research Communications*.

[30] Wu Q., Zhang J., Wan Y. (2015). Hydrogen water alleviates lung injury induced by one-lung ventilation. *Journal of Surgical Research*.

[31] Chen C., Chen Q., Mao Y. (2010). Hydrogen-rich saline protects against spinal cord injury in rats. *Neurochemical Research*.

[32] Li J., Hong Z., Liu H. (2016). Hydrogen-rich saline promotes the recovery of renal function after ischemia/reperfusion injury in rats via anti-apoptosis and anti-inflammation. *Frontiers in Pharmacology*.

[33] Corris P. A., Christie J. D. (2007). Update in transplantation 2006. *American Journal of Respiratory and Critical Care Medicine*.

[34] King R. C., Binns O. A., Rodriguez F. (2000). Reperfusion injury significantly impacts clinical outcome after pulmonary transplantation. *The Annals of Thoracic Surgery*.

[35] Kawamura T., Huang C.-S., Peng X. (2011). The effect of donor treatment with hydrogen on lung allograft function in rats. *Surgery*.

[36] Zhang J., Zhou H., Liu J., Meng C., Deng L., Li W. (2019). Protective effects of hydrogen inhalation during the warm ischemia phase against lung ischemia-reperfusion injury in rat donors after cardiac death. *Microvascular Research*.

[37] Zhou H., Fu Z., Wei Y. (2013). Hydrogen inhalation decreases lung graft injury in brain-dead donor rats. *The Journal of Heart and Lung Transplantation*.

[38] Tanaka Y., Shigemura N., Kawamura T. (2012). Profiling molecular changes induced by hydrogen treatment of lung allografts prior to procurement. *Biochemical and Biophysical Research Communications*.

[39] Kayawake H., Chen-Yoshikawa T. F., Saito M. (2021). Protective effects of a hydrogen-rich preservation solution in a canine lung transplantation model. *The Annals of Thoracic Surgery*.

[40] Saito M., Chen-Yoshikawa T. F., Takahashi M. (2020). Protective effects of a hydrogen-rich solution during cold ischemia in rat lung transplantation. *The Journal of Thoracic and Cardiovascular Surgery*.

[41] Liu R., Fang X., Meng C. (2015). Lung inflation with hydrogen during the cold ischemia phase decreases lung graft injury in rats. *Experimental Biology and Medicine*.

[42] Zhang G., Li Z., Meng C. (2018). The anti-inflammatory effect of hydrogen on lung transplantation model of pulmonary microvascular endothelial cells during cold storage period. *Transplantation*.

[43] Noda K., Shigemura N., Tanaka Y. (2014). Hydrogen preconditioning during ex vivo lung perfusion improves the quality of lung grafts in rats. *Transplantation*.

[44] Haam S., Lee S., Paik H. C. (2015). The effects of hydrogen gas inhalation duringex vivolung perfusion on donor lungs obtained after cardiac death. *European Journal of Cardio-Thoracic Surgery*.

[45] Haam S., Lee J. G., Paik H. C., Park M. S., Lim B. J. (2018). Hydrogen gas inhalation during ex vivo lung perfusion of donor lungs recovered after cardiac death. *The Journal of Heart and Lung Transplantation*.

[46] Meng C., Ma L., Niu L. (2016). Protection of donor lung inflation in the setting of cold ischemia against ischemia-reperfusion injury with carbon monoxide, hydrogen, or both in rats. *Life Sciences*.

[47] Kawamura T., Huang C.-S., Tochigi N. (2010). Inhaled hydrogen gas therapy for prevention of lung transplant-induced ischemia/reperfusion injury in rats. *Transplantation*.

[48] Ishikawa T., Shimada S., Fukai M. (2018). Post-reperfusion hydrogen gas treatment ameliorates ischemia reperfusion injury in rat livers from donors after cardiac death: a preliminary study. *Surgery Today*.

[49] Tamaki I., Hata K., Okamura Y. (2018). Hydrogen flush after cold storage as a new end-ischemic ex vivo treatment for liver grafts against ischemia/reperfusion injury. *Liver Transplantation*.

[50] Uto K., Sakamoto S., Que W. (2019). Hydrogen-rich solution attenuates cold ischemia-reperfusion injury in rat liver transplantation. *BMC Gastroenterology*.

[51] Shimada S., Wakayama K., Fukai M. (2016). Hydrogen gas ameliorates hepatic reperfusion injury after prolonged cold preservation in isolated perfused rat liver. *Artificial Organs*.

[52] Abe T., Li X.-K., Yazawa K. (2012). Hydrogen-rich University of Wisconsin solution attenuates renal cold ischemia–reperfusion injury. *Transplantation*.

[53] Kobayashi E., Sano M. (2019). Organ preservation solution containing dissolved hydrogen gas from a hydrogen-absorbing alloy canister improves function of transplanted ischemic kidneys in miniature pigs. *PLoS One*.

[54] Shigeta T., Sakamoto S., Li X.-K. (2015). Luminal injection of hydrogen-rich solution attenuates intestinal ischemia-reperfusion injury in rats. *Transplantation*.

[55] Yamamoto H., Aokage T., Igawa T. (2020). Luminal preloading with hydrogen-rich saline ameliorates ischemia-reperfusion injury following intestinal transplantation in rats. *Pediatric Transplantation*.

[56] Buchholz B. M., Masutani K., Kawamura T. (2011). Hydrogen-enriched preservation protects the isogeneic intestinal graft and amends recipient gastric function during transplantation. *Transplantation*.

[57] Buchholz B., Kaczorowski D., Sugimoto R. (2008). Hydrogen inhalation ameliorates oxidative stress in transplantation induced intestinal graft injury. *American Journal of Transplantation*.

[58] Nakao A., Kaczorowski D. J., Wang Y. (2010). Amelioration of rat cardiac cold ischemia/reperfusion injury with inhaled hydrogen or carbon monoxide, or both. *The Journal of Heart and Lung Transplantation*.

[59] Noda K., Shigemura N., Tanaka Y. (2013). A novel method of preserving cardiac grafts using a hydrogen-rich water bath. *The Journal of Heart and Lung Transplantation*.

[60] Tan M., Sun X., Guo L., Su C., Sun X., Xu Z. (2013). Hydrogen as additive of HTK solution fortifies myocardial preservation in grafts with prolonged cold ischemia. *International Journal of Cardiology*.

